# Immune reconstitution inflammatory syndrome in association with HIV/AIDS and tuberculosis: Views over hidden possibilities

**DOI:** 10.1186/1742-6405-4-29

**Published:** 2007-11-30

**Authors:** Esaki Muthu Shankar, Ramachandran Vignesh, Kailapuri G Murugavel, Pachamuthu Balakrishnan, Ramalingam Sekar, Charmaine AC Lloyd, Suniti Solomon, Nagalingeswaran Kumarasamy

**Affiliations:** 1 YRG Centre for AIDS Research and Education, VHS Hospital Campus, Rajiv Gandhi Salai – Information Technology Corridor, Taramani, Chennai 600 113, India

## Abstract

Gut immune components are severely compromised among persons with AIDS, which allows increased translocation of bacterial lipopolysaccharides (LPS) into the systemic circulation. These microbial LPS are reportedly increased in chronically HIV-infected individuals and findings have correlated convincingly with measures of immune activation. Immune reconstitution inflammatory syndrome (IRIS) is an adverse consequence of the restoration of pathogen-specific immune responses in a subset of HIV-infected subjects with underlying latent infections during the initial months of highly active antiretroviral treatment (HAART). Whether IRIS is the result of a response to a high antigen burden, an excessive response by the recovering immune system, exacerbated production of pro-inflammatory cytokines or a lack of immune regulation due to inability to produce regulatory cytokines remains to be determined. We theorize that those who develop IRIS have a high burden of proinflammatory cytokines produced also in response to systemic bacterial LPS that nonspecifically act on latent mycobacterial antigens. We also hypothesize that subjects that do not develop IRIS could have developed either tolerance (anergy) to persistent LPS/tubercle antigens or could have normal FOXP3+ gene and that those with defective FOXP3+ gene or those with enormous plasma LPS could be vulnerable to IRIS. The measure of microbial LPS, anti-LPS antibodies and nonspecific plasma cytokines in subjects on HAART shall predict the role of these components in IRIS.

## Background

### Immune reconstitution inflammatory syndrome (IRIS): An existing lacuna in HIV immunology?

IRIS is an adverse consequence of the restoration of pathogen-specific immune responses in HIV-infected patients during the initial months of highly active antiretroviral treatment (HAART) [1]. Even though IRIS is also closely associated with certain other infectious (mycobacteria, varicella zoster, herpesviruses, and cytomegalovirus) and non-infectious (autoimmune) conditions [2-10], the morbidity associated with HIV/tuberculosis (TB) is more important [1,11] as the crisis seem to be alarming in third-world nations, where the proportion of HIV/TB IRIS is reportedly high, ranging from 11% to 43% [12-15]. This could be due to differences in cohort characteristics, case definitions and differences in the mean time interval between TB diagnosis and antiretroviral therapy (ART) initiation. Data from resource-limited countries on TB-IRIS is scarce; a rate of 8% was reported from India [1]. Immunology of IRIS in HIV/TB is deficient and HIV-specific T lymphocyte responses have repeatedly shown to be defective [16]. To understand the immunopathogenesis of IRIS it will be crucial to elucidate the intrinsic dynamics of immune cells after initiation of HAART [17]. Preliminary investigations have shown that an acute exacerbation of mycobacteria-specific Th1 response after HIV infection control by HAART causes IRIS in HIV/TB [17,18].

### Does CD4+ T-cell depletion lead to a breach in gut immune cell integrity to initiate the proinflammatory cytokine saga?

In the context of an HIV infected subject with latent pulmonary TB, progressing to AIDS stage of HIV disease, the acute stage of the infection is characterized by eventual depletion in the number of CD4+ T-cells, the key orchestrator of all immune mechanisms in the body. Recent research has re-examined the rate of immunopathologic events in HIV disease, where the first few weeks is characterized by massive viremia and depletion of ~50% memory CD4+ T-cell (CCR5+) population especially in the gut [19-26]. Since the gut associated lymphoid tissue (GALT) comprises ~60% of entire lymphoid organ system, rich in memory cells, its depletion has a strong consequence on the entire CD4+ T-cell population. Memory CD4+ T-cells in the lamina propria is depleted principally by Fas-FasL-mediated cell death [26]. In addition, productive HIV infection is favored by an inflammatory environment, because Th1 cytokines (IL-2, IL-12, TNF-α) increase NFkB activation in T-cells, which drives HIV transcription. Early breach in the gut mucosal integrity and epithelial microenvironment [19-21,27-30] leads to increased translocation of luminal microbial products [20] because the gut is thought to be the principal source of microbial products (especially LPS) and because it has a massive bacterial load compared to other anatomical sites [31-33]. Translocation results in chronic inflammation via Toll-like receptor (TLR-4) stimulation, resulting in cytokine and chemokine release driving persistent T-cell activation and (tat mediated) apoptosis via activation-induced cell death (AICD) [21]. However, due to lack of sufficient CD4+ T-cells, complex inflammatory mechanisms might not be expected due to anergy.

### HAART, immunological restoration and the inflammatory milieu: Who are the possible mediators?

Most of the subjects with HIV disease attend HIV testing centers in India only after advanced clinical HIV disease (AIDS) sets in and when their CD4+ T-cell counts are low [1,11]. In spite of initiation of HAART, some experience a 'discordant response', whereby the HIV-1 RNA plasma level is below the limit of detection but the CD4+ cell count response is blunted. We propose that these individuals with HIV/TB coinfection might not progress to clinical IRIS owing to poor immune reconstitution despite considerable virological recovery. As a consequence, a substantial proportion of treated individuals show poor CD4+ T-cell recovery [40]. This has also been correlated with a lower nadir pretreatment CD4+ T-cell count, suggestive of more extensive depletion of CD4+ T-cells in the GALT during acute HIV infection, which may be refractory to reconstitution with ART [19,41]. Initiation of HAART allows 'partial' immune restoration [42], which however, can result in the substantial proliferation and differentiation of most of the immune components [43,44]. Due to immune restoration, an inflammatory response against infectious and non-infectious antigens (LPS) is mounted leading to noticeable 'paradoxical worsening' [43-45], with a shift toward a Th1 receptor profile, which increases the levels of IFN-γ and IL-2 [46-51]. Therefore, persons with latent TB or other systemic commensal antigens (LPS) could lead to exaggerated inflammatory responses. Studies also show that an inflammatory response is required for the elimination of any gram-negative infection (i.e. LPS) [52]. HAART treatment (that enable 'partial' immune reconstitution) considerably reduces circulating LPS although total clearance may not be feasible for considerable periods of time.

Bacterial LPS, the microbe-associated molecular patterns (MAMP) of gram-negative bacteria are known potent activators of cells of inflammatory system. Plasma LPS levels have been directly associated with the degree of intestinal permeability following invasive gastrointestinal surgery [34], inflammatory bowel disease (IBD) [35] and graft-versus host disease (GVHD) [31,35-39]. Experimental SIV infection of macaques resulted in raised circulating LPS levels [21]. Recent studies have found significantly elevated levels of plasma LPS in chronically HIV-infected humans with progressive disease [21] and has correlated convincingly with measures of innate and adaptive immune activation. Besides, the study also has shown the association between LPS and chronic *in vivo *stimulation of monocytes, an association between raised plasma LPS; and an association between reduction in plasma LPS and CD4+ T-cell reconstitution with HAART [21]. Due to abrupt increase in the numbers of CD4+ T-cells, the pattern recognition receptors (PRR) induce signal transduction pathway molecules like NFkB, IL-1 receptor, TNF receptor, MAP kinase receptor etc. [53]. Cytokines such as IL-1 can also stimulate the NFkB binding molecule to activate NFkB [54-56], which induces the expression of cyclooxygenase-2 (COX-2), which consequently leads to tissue inflammation at the site where latent TB antigens are located. Interestingly, the expression of the COX-2-encoding gene, believed to be responsible for the massive production of prostaglandins at inflammatory sites, is transcriptionally regulated by NFkB [54]. NFkB resides in the cytoplasm and is bound to its inhibitor. Furthermore, injurious and inflammatory stimuli, such as free radicals present in the plasma of the immune deteriorated host leads to NFkB release that subsequently moves into the nucleus to activate the genes responsible for COX-2 expression.

**Figure 1:**
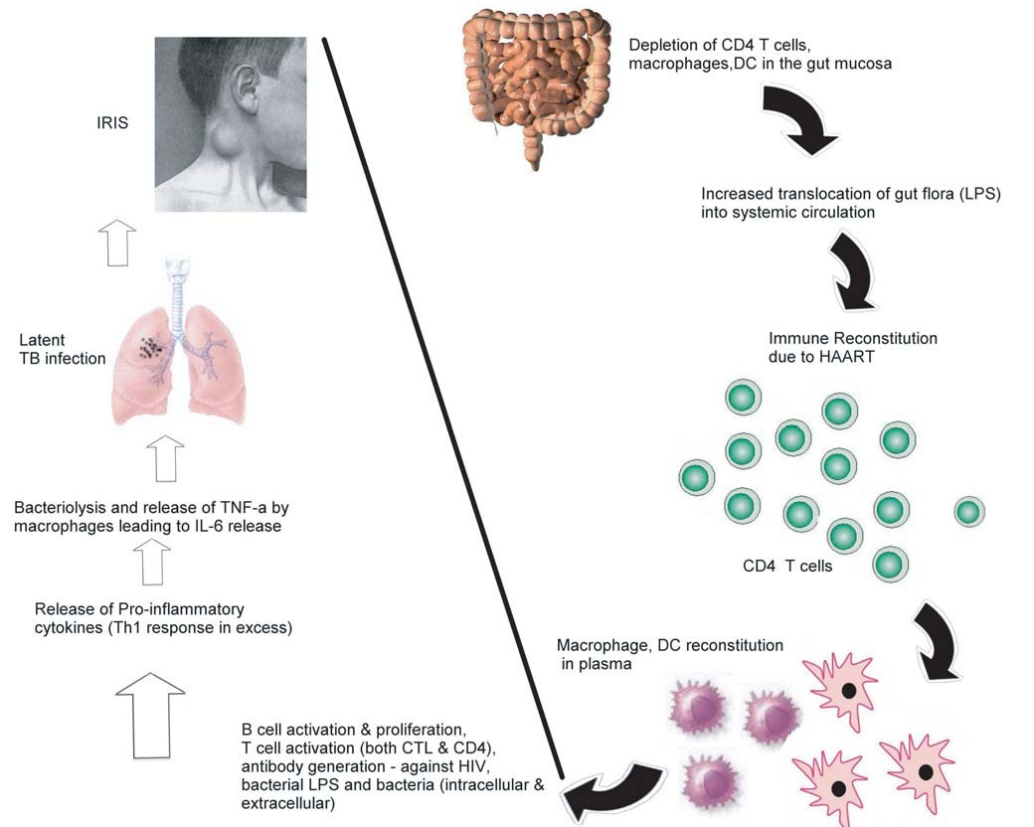
**One possible mechanism that illustrates the immunology of IRIS in a subject with HIV/TB coinfection**. Compromised gut immunity leads to increased translocation of luminal gram negative bacterial LPS into the systemic circulation. Initiation of HAART in the subject leads to abrupt restoration of CD4+ T-cells and almost any pathogen-specific immune response. IRIS developers have a high burden of LPS and proinflammatory cytokines produced against LPS could result in an exaggerated, nonspecific attack on latent mycobacterial antigens that are presented in the local lymph nodes leading to localized inflammation. We also hypothesize that subjects that do not develop IRIS could have developed either tolerance (anergy) to persistent LPS and tubercle antigens or could have normal FOXP3+ gene (not shown) and that those with defective FOXP3+ gene or enormous plasma LPS could be vulnerable to IRIS (as demonstrated by researchers that defective FOXP3+ gene is associated with increased risk for inflammatory conditions). (Bold lines indicate the availability of clinical/experimental evidence and dashed lines indicate the possible mechanism).

Alternatively, effector T-cells of the Th1 subset activates macrophages by CD154 – CD4+0 interactions and by secreting IFN-γ. Th1 subsets produce the proinflammatory cytokines, IL-2, IFN-γ, and TNF-α, and Th2 cells, the anti-inflammatory cytokines, IL-4, 5, 6, 10, and 13. In addition, macrophages that have phagocytosed TB bacilli produce IL-12 that stimulates the differentiation of naïve CD4+ T-cells to the Th1 subset, which again produces IFN-γ on encountering macrophage-associated microbial antigens; IL-12 also increases the amount of IFN-γ produced by these T-cells. In different T-cell mediated diseases, tissue injury is caused by a delayed-type hypersensitivity response mediated by CD4+ T-cells or by lysis of host cells by CD8+ CTLs. Some studies suggest that circulating IL-6 levels prior to HAART may be associated with IRIS [53]. CD4+ T-cells may react against cell or tissue antigens and secrete cytokines that induce local inflammation and activate macrophages. The actual tissue injury is caused by the macrophages and other inflammatory cells. CD8+ T-cells specific for antigens on autologous cells may directly kill these cells. Increased LPS-binding protein (LBP) may also increase the host response and potentiate injury. We hypothesize that the excessive presence of LPS in HIV/TB coinfected subjects accounts for the progression of IRIS and those that have LPS in limited concentrations may not. In studies in which normal human subjects were treated with LPS intravenously, there was a shift toward a Th2 response with increased expression of IL-10, [57-59] and the pretreatment of healthy human volunteers with IL-10 reduced the LPS-induced increases in chemokines [60,61]. Data from studies in normal human volunteers suggest that LPS increase the production of circulating IL-10, which would then blunt the proinflammatory response to a second bacterial challenge [60,61]. The Th2 shift in sepsis suggests that an excess of anti-inflammatory cytokines may result in impaired lung host response. We therefore hypothesize that this situation could also lead to extensive multiplication of TB bacilli. A brief overview of the concept is illustrated in figure 1.

### The likelihood of 'normal' FOXP3+ gene and endotoxin-tolerance among IRIS non-developers – Why?

This 'paradoxical worsening' could also be attributable to additional presence of defective FOXP3+ gene among IRIS developers. Presence of defective FOXP3+ gene in T-cells has been reported to confer increased risk of inflammatory conditions in human beings in contrast to a normal FOXP3+ gene. After an initial exposure to LPS, monocytes and macrophages become refractory to subsequent LPS challenge (endotoxin-tolerance) (57 – 61). This initially was believed to be protective against septic shock. However, recent evidence suggests that endotoxin-tolerance impairs the host response to a second bacterial challenge [62,63]. The prolonged presence of TB antigens (and a normal FOXP3+ gene) could also lead to anergy and poor immune responses to TB antigens despite HAART. Monocytes obtained from septic patients have functional defects that include profound defects in IL-1, 6, and TNF-α production; loss of HLA class II antigen expression; and impaired antigen presentation [64-69]. In patients with sepsis, monocytes from survivors showed normal cytokine response following LPS stimulation [64]. A potential mechanism whereby endotoxin-tolerance develops is a down-regulation of LPS receptors such as membrane CD14 on macrophages [70]. The exposure of monocytes and macrophages to the anti-inflammatory cytokines, IL-10 and TGF-β, is a second mechanism that may be responsible for the monocyte deactivation that resembles endotoxin-tolerance [71]. Studies performed with human alveolar macrophages exposed to IL-10 *in vitro *show increased intracellular bacterial replication of *Legionella pneumophila*, [72] and decreased production of proinflammatory cytokines [73]. These suggest that macrophages and monocytes in septic patients may develop a phenotype similar to that observed in endotoxin-tolerance, which could result in an impaired response to lung pathogens. The development of tolerance was hypothesized to be beneficial by diminishing the proinflammatory response in patients with sepsis. However, some data suggest that the development of tolerance may worsen clinical outcomes because monocytes and macrophages may not respond adequately to a bacterial challenge [62,65,74]. The CD14/TLR complex and associated signaling pathways are essential for the recognition of LPS by macrophages, and several studies suggest that down-regulation of CD14/TLR complexes on macrophages is responsible for the development of tolerance [63,70,75,76]. However, the development of tolerance does not correlate with down-regulation of LPS-binding sites [77], suggesting the possible role of other mechanisms including the disruption of CD14/TLR signaling pathways [78] and the macrophage exposure to anti-inflammatory IL-10 [79]. Therefore, it is hypothesized that subjects that do not progress to develop IRIS (IRIS tolerant) despite HAART initiation could develop tolerance (anergy) to persistent LPS/tubercle antigens.

## Conclusion

It is hypothesized that proinflammatory cytokines produced excessively in response to systemic bacterial LPS nonspecifically act on latent mycobacterial antigens leading to clinical deterioration and 'paradoxical worsening' of inflammatory responses against both infectious (HIV/TB) and non-infectious (LPS) microbial antigens. This 'paradoxical worsening' could also be attributable to additional presence of defective FOXP3+ gene among IRIS developers. Subjects that do not progress to develop IRIS (IRIS tolerant) despite HAART initiation could develop either tolerance (anergy) to persistently existing LPS and tubercle antigens. Thus far, no single treatment option exists against IRIS and depends on the underlying infectious agent and its clinical presentation. However, since the pathogenesis is an inflammatory one, systemic corticosteroids or non-steroidal anti-inflammatory drugs (NSAIDS) may assuage the symptoms. Therefore, studies must be attempted to assess the role of immunological correlates and possible markers of IRIS needs to be evaluated to better understand the mechanisms behind IRIS in HIV/TB or other opportunistic coinfections, which would largely facilitate the timely management of IRIS in HIV/AIDS.

## Competing interests

The author(s) declare that they have no competing interests.

## Authors' contributions

EMS, RV and NK conceived and proposed the hypothesis. RV, KGM, PB, CAL, RS, SS, and NK provided additional inputs to further develop the scientific concept; EMS, RV and PB drafted the manuscript; SS and NK shared their clinical expertise and critically revised the manuscript. All authors read and approved the final manuscript. EMS, RV and NK are the guarantors of the paper.
